# UAVs, Hyperspectral Remote Sensing, and Machine Learning Revolutionizing Reef Monitoring

**DOI:** 10.3390/s18072026

**Published:** 2018-06-25

**Authors:** Mark Parsons, Dmitry Bratanov, Kevin J. Gaston, Felipe Gonzalez

**Affiliations:** 1 Queensland University of Technology, 2 George St, Brisbane, QLD 4000, Australia; 2 Research Engineering Facility, Institute for Future Environments, Queensland University of Technology, 2 George St, Brisbane, QLD 4000, Australia; Dmitry.Bratanov@qut.edu.au; 3 Environment and Sustainability Institute, University of Exeter, Penryn, Cornwall TR10 9FE, UK; k.j.gaston@exeter.ac.uk; 4 Institute for Advanced Study, Wissenschaftskolleg zu Berlin, Wallotstrasse 19, 14193 Berlin, Germany; 5 Institute for Future Environments, Robotics and Autonomous Systems, Queensland University of Technology, 2 George St, Brisbane, QLD 4000, Australia; felipe.gonzalez@qut.edu.au

**Keywords:** in-water survey, UAS, hyperspectral camera, machine learning, image segmentation, support vector machines (SVM), drones

## Abstract

Recent advances in unmanned aerial system (UAS) sensed imagery, sensor quality/size, and geospatial image processing can enable UASs to rapidly and continually monitor coral reefs, to determine the type of coral and signs of coral bleaching. This paper describes an unmanned aerial vehicle (UAV) remote sensing methodology to increase the efficiency and accuracy of existing surveillance practices. The methodology uses a UAV integrated with advanced digital hyperspectral, ultra HD colour (RGB) sensors, and machine learning algorithms. This paper describes the combination of airborne RGB and hyperspectral imagery with in-water survey data of several types in-water survey of coral under diverse levels of bleaching. The paper also describes the technology used, the sensors, the UAS, the flight operations, the processing workflow of the datasets, the methods for combining multiple airborne and in-water datasets, and finally presents relevant results of material classification. The development of the methodology for the collection and analysis of airborne hyperspectral and RGB imagery would provide coral reef researchers, other scientists, and UAV practitioners with reliable data collection protocols and faster processing techniques to achieve remote sensing objectives.

## 1. Introduction

Coral reefs are under pressure, and as of 2016, 34.8% of the Great Barrier Reef has been affected by coral bleaching, and other reefs worldwide are also experiencing degradation due to industrial and environmental events (e.g., cyclones) [1]. Large scale monitoring of coral reefs is an expensive, time-consuming, and challenging task, which utilises various mediums (satellite, airplane, boat and both manned and unmanned in-water surveys). Current surveillance practice for reef monitoring is to use satellites, ship-based multibeam sonar (MBES), plane based airborne surveying, or visually inspecting for signs of bleaching damage [2,3]. Over the last decades, the commercial satellite-based remote sensing capability has been significantly improved [4]. Now, with the best ground sampling distance (GSD) of 30 cm for panchromatic, and 1.2 m for multispectral data, satellite imagery remains the best-suited option for large-scale monitoring of entire reef. The satellite surveys, however, have some significant limitations, due to cloud cover and to camera resolution hindering the ability to distinguish finer details of reef ecosystems. Indeed, the approach struggles to provide marine researchers and biosecurity managers with often required benthic conditions on the sub-centimetre level GSD.

Recent advances in remote sensed imagery and geospatial image processing using unmanned aerial systems (UASs) can enable rapid and ongoing monitoring tools for coral bleaching detection and surveillance. This paper describes a UAS-based remote sensing methodology to increase the efficiency of existing surveillance practices, to detect coral type and coral bleaching, by combining in-water spectral observations and automated classification techniques to determine levels of benthic cover and coral growth in shallow water reef regions. The methodology is evaluated on its ability to identify differences between coral genera, bleaching level, and water depth, with an in-depth accuracy assessment to give insights for further refinements. This shows that UAS-based hyperspectral remote sensing techniques can offer an efficient and cost-effective approach to mapping and monitoring reef habitats over large, remote, and inaccessible areas.

The technology used, the sensors, the UAV, and the flight operations are presented in Section 2 of the paper. Later, in Section 3, we discuss the processing workflow for each imagery dataset, and methods for combining multiple airborne with ground-based datasets. Section 4 presents results of correlation between the different processed datasets.

## 2. Materials and Methods

**Figure 1:**
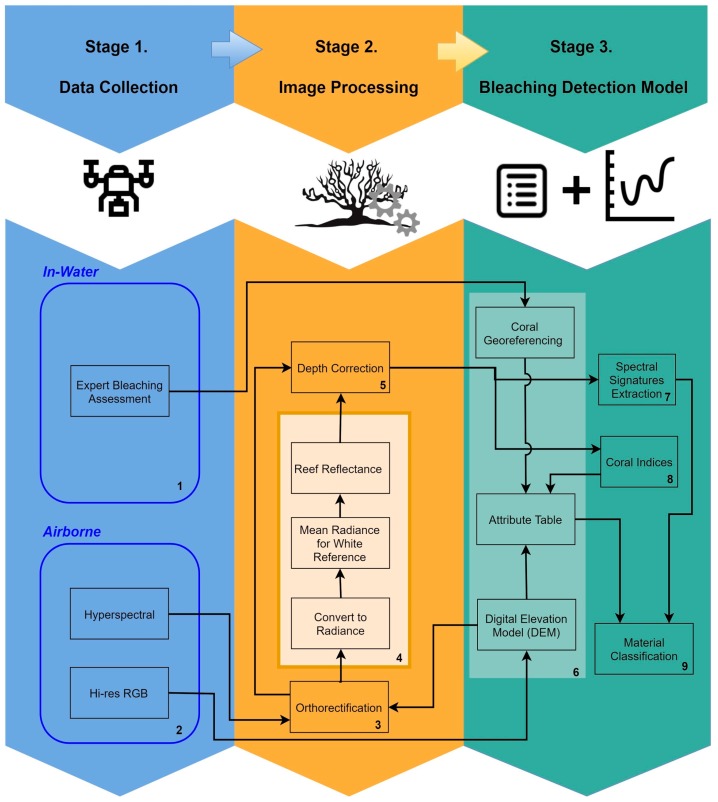
Predictive detection model workflow.

Our approach to generate a predictive reef monitoring system employs multiple stages (Figure 1). The first is data collection. This includes collection of airborne RGB and hyperspectral imagery, as well as in-water expert visual assessment of each coral specimen.

In the second stage, images are processed (using data in Table 1) to obtain the coral type, bleaching assessment, and reef orthomosaics. Hyperspectral scans are then orthorectified using multiple datacubes to produce a georeferenced hyperspectral snapshot of the reef. These orthorectified datacubes are processed to obtain radiance and reflectance, that are used in combination with expert visual coral type and bleaching assessment for signature extraction and calculation of indices of coral types and bleaching levels.

The third stage combines all the multiple sources of data into a single information system. To do this, individual coral genera are segmented, with the aim of creating a table (Table 2) that contains different attributes for each coral specimen within the reef.

1. Coral genus
2. Expert bleaching assessment (1 to 6 where 1 relates to severely bleached coral, 6—unbleached)
3. Depth
4. Notes about surroundings (e.g., coral size, proximity to other coral)
5. Latitude
6. Longitude

Once the table is populated with georeferenced data, the spectral signatures are then extracted to generate a correlation between coral genus and each bleaching level. Results obtained in the correlation analysis are the foundation for the development of a preliminary coral bleaching detection model, which is followed by an evaluation and accuracy assessment to obtain final reef belching detection model.

### 2.1. In-Water Surveys

Coral is categorised by the Australian Institute of Marine Science (AIMS) into one of six levels of bleaching through visual inspection, where level one is severely bleached and level six an unbleached healthy coral specimen. Due to water’s optical properties and algal growth associated with reef ecosystems, the spectral properties of a coral sample can vary drastically based on the current conditions, genus, and depth of the area.

**Figure 2:**
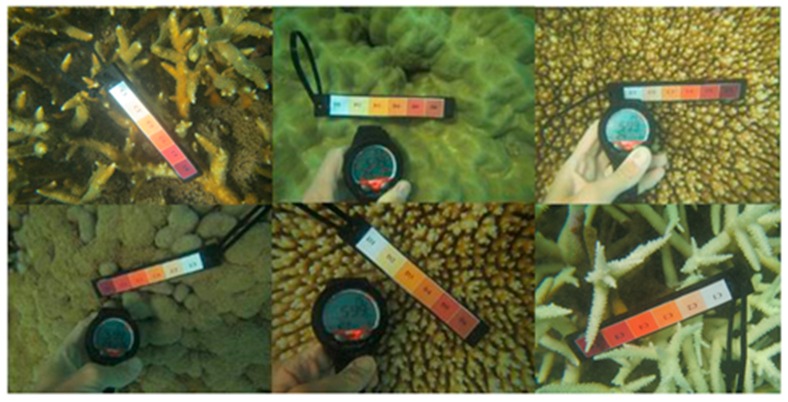
Example of in-water reference data with relevant bleaching level: 1 (severely bleached) to 6 (unbleached). Bleaching levels and genus from top left to bottom right: 5 *Acropora*, 4 *Porites* massive, 3 *Acropora*, 3 *Goniopora*, 2 *Acropora*, 1 *Acropora*. Courtesy of Sam Noonan (Australian Institute of Marine Science (AIMS)).

**Figure 3:**
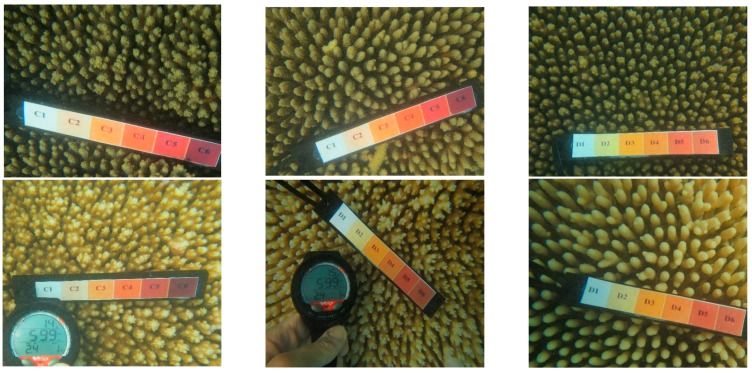
Example of in-water reference data of *Acropora* with relevant bleaching level: 1 (severely bleached) to 6 (unbleached). **Top left**—6, **bottom right**—1. Courtesy of Sam Noonan (AIMS).

Our approach was to use survey test sites where experts identified various genera of coral at varying levels of bleaching across a segment of reef, as a reference for approximation of optical variances due to water and algae. Figure 2 and Figure 3 show examples of coral bleaching data obtained at different in-water survey locations, and after each point is identified, they are then classified based on genus, location (longitude and latitude), depth, and bleaching level.

### 2.2. Airborne Methods, UAV, and Sensors

The UAV used was an S800 EVO Hexacopter (DJI-Innovations, Shenzhen, China) that weighs 6.0 kg with motors, propellers, electronic speed controllers (ESCs), control and navigation avionics, and 16,000 mAh battery. The frame is fitted with a retractable undercarriage, providing a sensor field of view clear of obstacles. The UAV has a recommended maximum take-off weight of 8 kg, thus allowing 2 kg for sensor payload. A WooKong-M flight controller provides the navigation and control systems of the UAV, and comes with a stabilization controller and GPS unit with inbuilt compass and inertial management unit (IMU). The flight controller has multiple autopilot modes to enable both remote control by operator and autonomous with position and altitude hold, and auto go home/landing with enhanced fail-safe.

In this work and methodology, we propose the use of high resolution RGB and hyperspectral UAV-based imagery, as they provide the required level of detail in capturing finer differences in coral bleaching signatures. A Canon EOS 5DS R (Canon, Tokyo, Japen) digital camera was used to capture high-resolution RGB images from the mission route, and assist in identification and monitoring of coral in the studied area using the imagery augmented by the camera’s GPS coordinates. The camera specifications include full frame (36 × 24 mm) CMOS 50.6-megapixel sensor, 28-mm Canon lens, and a single-frequency GPS receiver. 

Hyperspectral imagery was acquired using a Headwall Nano-Hyperspec (Headwall Photonics Inc., Bolton, MA, USA) hyperspectral sensor. This sensor records datacubes of 274 spectral bands in the VNIR (Visible and Near-Infrared) range (400–1000 nm) with ~2.2 nm spectral interval and 5 nm spectral resolution (fwhm with 20 μm slit). This sensor is equipped with a calibrated f/1.8 4.8 mm Schneider lens, which results in 50.7 deg field of view over 640 pixels. The collected hyperspectral data cubes are synchronized with GPS/INS (Inertial Navigation System) positioning and orientation information to perform datacube orthorectification.

**Figure 4:**
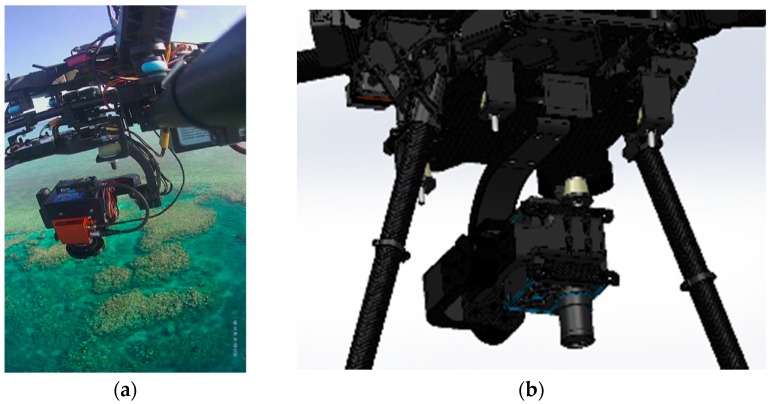
(**a**) Headwall Nano hyperspectral sensor onboard S800 UAV; (**b**) Custom-made 2-axis gimbal hosting the hyperspectral camera (SolidWorks 3D model).

Image stabilization is also an important step, therefore, the hyperspectral sensor was integrated into a S800 UAV by a custom designed gimbal made by Queensland University of Technology (QUT) Research Engineering Facility (REF). This gimbal has 2-axis stabilization, that ensures seamless operation of the push-broom hyperspectral scanner in windy conditions. The gimbal design features carbon reinforcement over 3D printed structure, advanced dampening, brushless motors, and a BaseCam SimpleBGS 32 bit gimbal controller (BaseCam electronics, Riga, Latvia), with the total weight below 1 kg. Mounting the push-broom scanner on the gimbal proved to enhance camera performance in wind gust conditions by ensuring the minimal and consistent required overlaps between consecutive datacubes over the large study area. This leads to an increased overall flight efficiency in real life open environments. Figure 4a shows the S800 UAV with the hyperspectral sensor on the gimbal during one of the missions. The gimbal CAD design model is presented in Figure 4b.

### 2.3. Orthorectification of Hyperspectral Images

**Figure 5:**
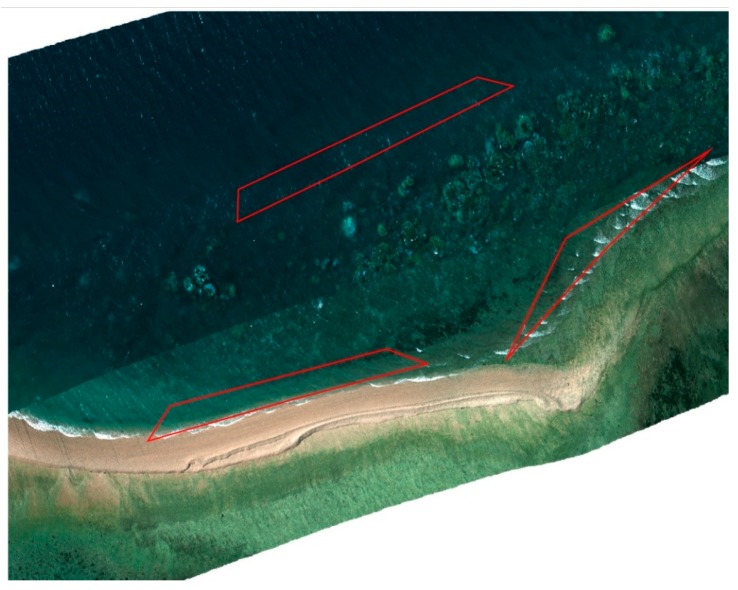
Multi-orthorectification by Headwall SpectralView overlaid in Google Earth, polygonal regions (in red) indicate visible spume regions in close proximity to coral.

Data orthorectification (step 3) was conducted using Headwall SpectralView software, where the individual hyperspectral data cubes are geolocalised and stitched, with verification by the overlay of underwater contours of the reef. Figure 5 shows stitched images overlaid in Google Earth.

The hyperspectral data are then processed with spectral analysis tasks, such as MATLAB Scyllarus open source toolbox, Headwall SpectralView, and Scyven, to obtain radiance values which are then processed with a white reference image to generate the reflectance dataset (step 4). Once obtained, the reflectance dataset is corrected for water depth by running through a noise reduction algorithm (step 5), processed through Scyven using support vector machine automated material discovery, to cluster similar spectral signatures which are geo-referenced (step 6), before being extracted (step 7) for reef indices (step 8) and material classification (step 9).

### 2.4. Image Processing, Radiance, Reflectance, White Reference

Step 4 orthorectified images were processed with Headwall SpectralView to identify the corresponding per pixel illumination value. Once completed, the image then goes through a semi-automated process in which the image is imported into Scyven, matched with a corresponding white reference illumination pattern (generated from white reference file), then lastly processed to generate the reflectance value corresponding to the white reference.

### 2.5. Depth Correction

**Figure 6:**
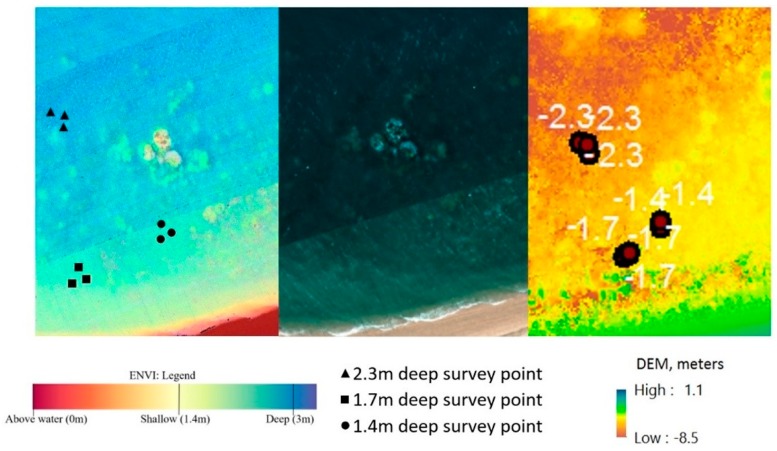
Depth approximation non-homogeneous composition (**left**—ENVI depth approximation; **centre**—Figure 5 extract; **right**—Agisoft Photoscan DEM (Digital Elevation Module) depth approximation).

Estimating and correcting hyperspectral imagery for water depth (step 5) is a complex process. ENVI offers unique tools for processing characteristics of waterbody features, and this enables quick and easy depth approximation utilizing a bottom albedo-independent bathymetry algorithm that utilizes a log ratio transformer. The log ratio transformer calculates depth independent of the bottom material and brightness (bright sand or dark seabed vegetation). The use of the algorithm means that typical errors caused by varying floor composition [5] (Figure 6) will not affect the overall accuracy of the approximation.

The use of additional information from in-water surveys is required for refining and generating accurate depth results with a reasonable degree of error. Agisoft Photoscan gives an alternative approach to the water depth estimation by using the reconstruction of a DEM from multiple overlaying images, which allows depth approximation of larger areas with ease, but comes with a more limited result set where smaller depth changes can be missed.

**Figure 7:**
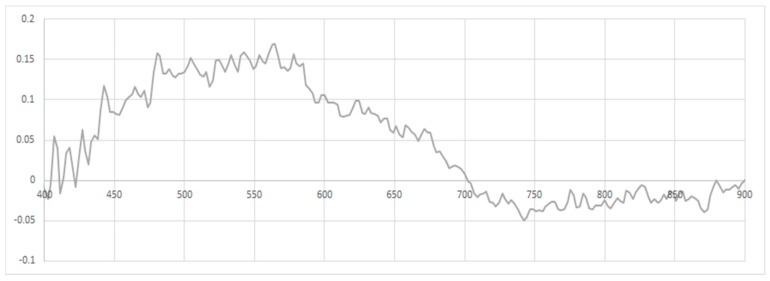
Spectral variance between massive *Porites* coral samples with level 4 bleaching at depths of 1 m and 2.3 m.

To apply depth correction, the effects of the water column must be ascertained, and to do this, the coral samples at varying water levels are compared; Figure 7 shows the variances in spectral signatures between two samples of massive *Porites* with a bleaching level of four, with one at a depth of 1 m and the other at 2.3 m.

**Figure 8:**
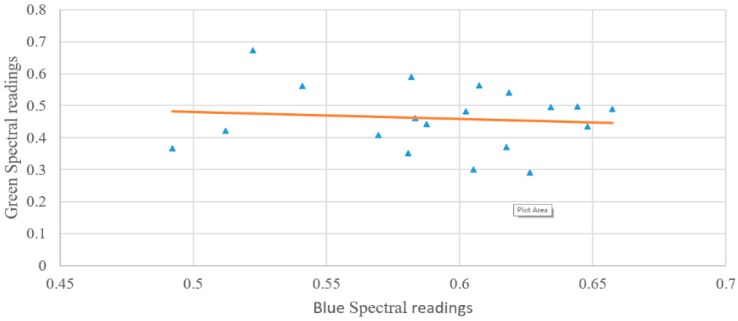
Spectral signature comparison between reflectance in blue and green bands of coral in different locations. Dots represent the ratio values between the green and blue spectral values, the orange line represents the trendline between all the data points.

To derive the effect of the water column on the data, the log-transformed ratio between the green and blue wavelengths is used, otherwise, the effects of varying benthic cover are also taken into account [6]. Once the data are derived, it can be seen that this is tightly packed (Figure 8).

This data is log transformed, and used as input into the equation identified by Mishra, D. [7] (below), which outputs the percentage variance due to water column effects.

(1) $$y=-18.353x^{2}+10.805x+0.238$$

This identified that 6.4% max variation in the spectral signature was due to water column effects, which could be corrected using a noise reduction algorithm, and follows the trend which was identified by Zoffoli, M [8].

### 2.6. Coral Georeferencing

**Figure 9:**
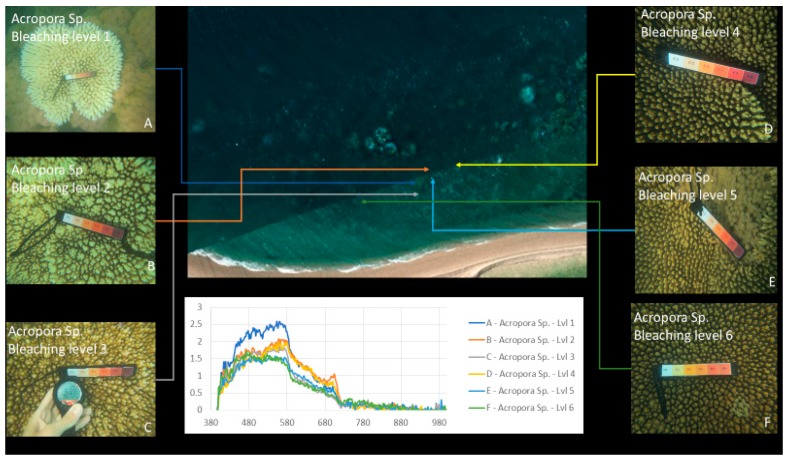
Examples of spectral responses “fingerprints” of bleaching levels 1 to 6 for *Acropora* coral.

In-water survey data contain GPS coordinates that are then matched to imagery. This can be completed by using the orthorectified data to overlay GPS coordinates across the image, and then extrapolating the corresponding pixel coordinates; Figure 9 shows an example of extrapolated coral locations and signature extraction. This data is then stored in an attribute table (step 6), along with the depth data and other characteristics (see Table 2 for table extract).

### 2.7. Spectral Signature Extraction

Expert visual assessment and georeferencing (step 6) are used with the depth-corrected reflectance values (step 5) to extract the mean spectral signature for different corals with different levels of bleaching. Headwall SpectralView is used to identify pixels that contain corals of varying bleaching levels, for which the spectral values are then extracted with Scyven. Figure 9 demonstrates the survey points for spectral signature extraction once the images have been converted into reflectance (step 4), then preprocessed with an automated material discovery (part of the material classification phase, step 9) which groups objects of similar spectral signatures.

After each genus of coral has been identified using material discovery, they then need to be compared based on their spectral signatures to identify potential issues with material classification (e.g., spectral signatures of each bleaching level not discernible for other genus samples).

**Figure 10:**
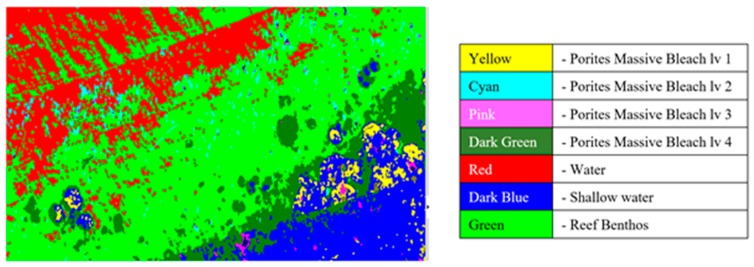
Coral identified with various levels of bleaching.

Figure 10 shows an example of the processed results where different regions are identified and grouped based on their spectral signatures. Brighter colours indicate regions with spectral patterns similar to that of the coral, sand, or other material identified in the region.

### 2.8. Reef Indices

**Figure 11:**
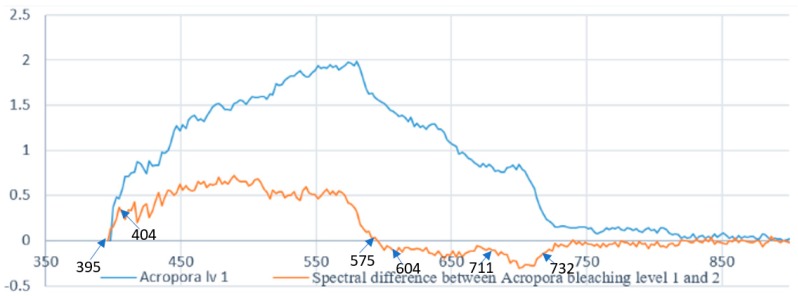
Spectral signature comparison for reef index generation Alv1.

The next step (step 8) is to calculate coral bleaching indices using the spectral signature extraction (step 7), as well as visual assessment (step 1), and georeferencing (step 6). The indices were selected to evaluate symptoms of bleaching such as higher spectral concentrations in the 400 nm and 750 nm regions. A list of the indices used, as well as their equations, is found in Table 1, where each genus of coral has been identified and then classified, based on differences in spectral samples comparing to the next closest spectral sample. Figure 11 shows an example of the spectral signature comparison used for coral bleaching index generation. 

### 2.9. Classification

Classification is the next step, where the in-water survey data along with the spectral signature extraction are combined using ENVI to develop pixel-by-pixel analysis algorithms, and also, subpixel (spectral mixture analysis) approaches using support vector machine (SVM). SVM was chosen due to the high classification accuracy, ability to handle multiple predictor variables, in-built handling of cases where spectral samples cannot be completely separated, and its ability to handle large datasets. It has also been found that the accuracy of SVM could be further increased if the gamma of images is increased up to a maximum of 75% (variations between captured data brightness and ideal gamma level are expected). This change causes SVMs to be equivalent to naive Bayes classifiers; Zanaty [9] proposed the SVM kernel of Gaussian radial basis polynomials function, which was found to be nearly 10% more accurate than the standard radial bias SVM classification method. Even without this, SVM is equivalent to the multilayer perception neural network in terms of accuracy of result. SVM using radial bias algorithm (shown below in Equation (2)) is therefore the method used in this paper, within the ENVI system, for material classifications.

(2) $$Radial\;Basis\;Function:\;K\left(x_{i},x_{j}\right)=exp\;\left(-\gamma {\left|\left|x_{i}-x_{j}\right|\right|}^{2}\right),\gamma >0$$

Scyven is also used to apply various noise reduction algorithms and data refinement through targeted unsupervised classification, to identify regions requiring additional accuracy refinements.

## 3. Field Experiments

### 3.1. Site and In-Water Surveys

**Figure 12:**
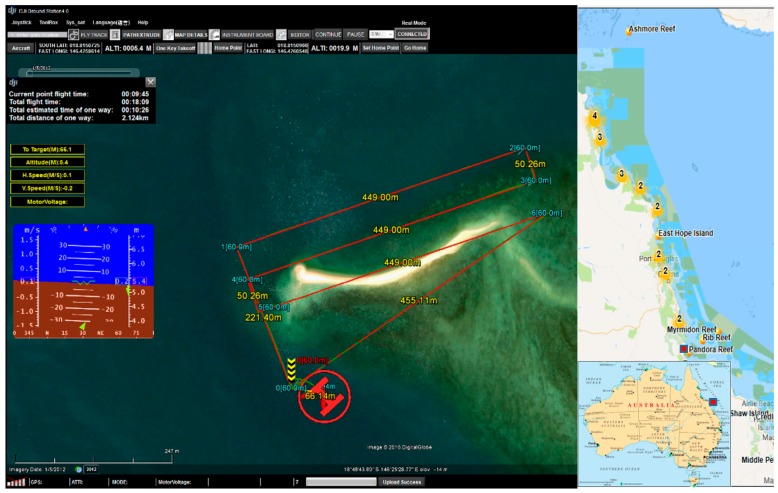
Pandora Reef site, Great Barrier Reef, QLD, Australia.

The site surveyed on 15 March 2017 is at Pandora Reef located in Queensland, Australia (Figure 12). The Australian Institute of Marine Science (AIMS) monitors this site regularly, and advised its selection due to the diverse range of coral genera, bleaching levels, and varying water depths. This paper demonstrates the method for an area of Pandora Reef in which 64 in-water survey points were collected to act as ground truths for spectral signature extraction (step 7) and training for the material classification (Table 2). 

### 3.2. In-Water Data Collection Survey

**Figure 13:**
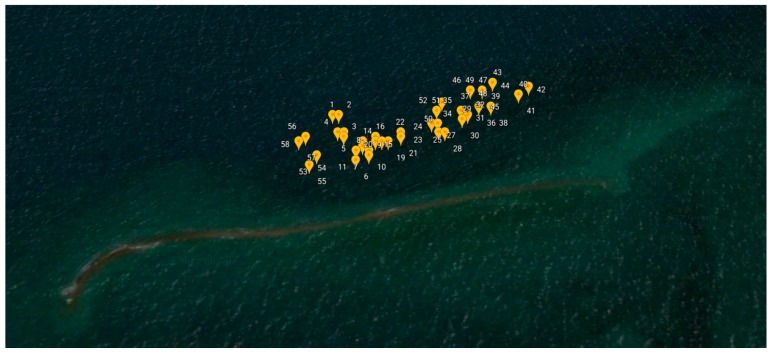
Numbered in-water survey GPS coordinates at Pandora Reef shown through Google Earth.

Table 2 summarizes the data which were collected from the in-water surveys on the 15 March 2017 at Pandora Reef. The following attributes were collected: date, reference image id, coral genus, bleaching level, depth, and additional notes about surroundings. These points were then mapped onto the orthorectified data, where its respective location is stored as a set of pixel coordinates. Figure 13 shows all the in-water survey locations mapped in Google Earth.

## 4. Results

### 4.1. Water Depth and Water Depth Extraction Results 

Figure 6 shows the result from ENVI’s relative water depth tool. Application of the bathymetry method is able to determine relative water depth by utilizing a log ratio transform. Visual assessment shows that the results in Figure 6 are consistent with the researcher’s knowledge of the study area and the in-water surveys (Figure 3).

#### 4.1.1. Orthomosaics

**Figure 14:**
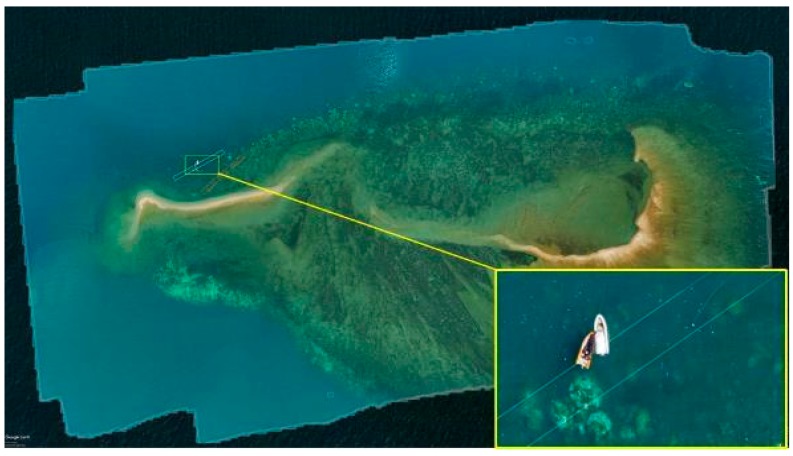
Photogrammetry orthomosaic of Pandora Reef (63 Ha) shows areas of day long in-water surveys performed by AIMS at the depth of 3 m (orange area) and 6 m (white area) 400% zoomed fragment on the bottom right (tide height 2.6 m).

The orthomosaic of the entire Pandora Reef (Figure 14) assisted in water depth extraction and generic reef structure. The orthomosaic was generated from Phantom 3 Pro images and is a geospatial RGB aerial view of the reef, with 5.71 cm/px resolution in a format of 234 megapixel image over 62.3 Ha area. The area was covered within a 20 min flight. Approximate areas covered by the underwater surveys are highlighted by red (3 m depth, 0.1 Ha) and white (6 m depth, 0.1 Ha) polygons.

**Figure 15:**
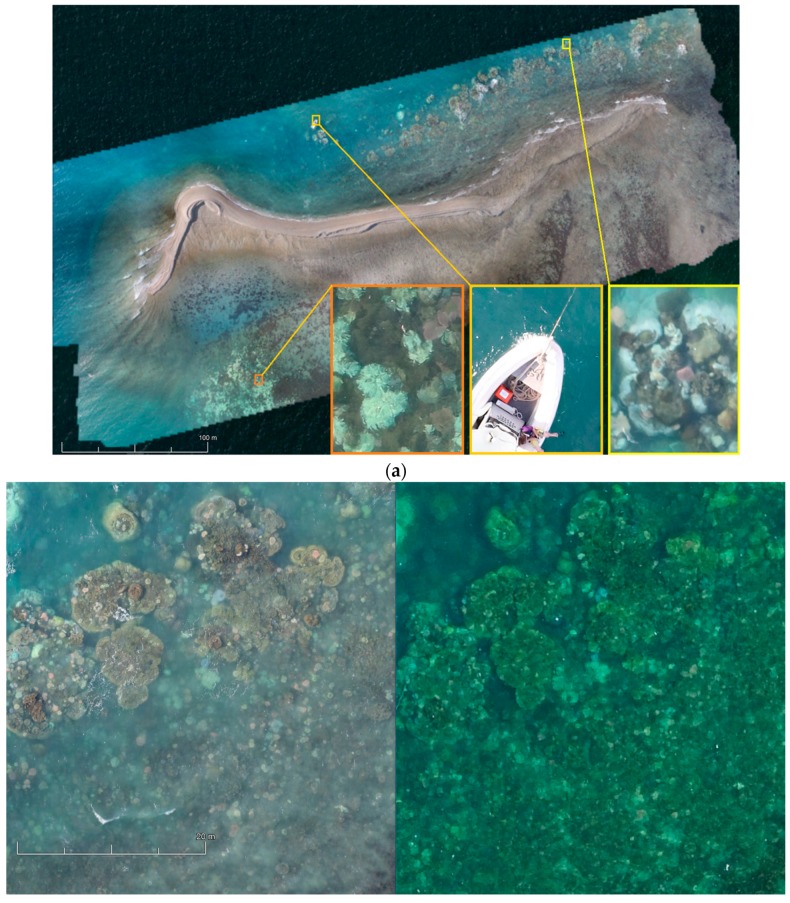

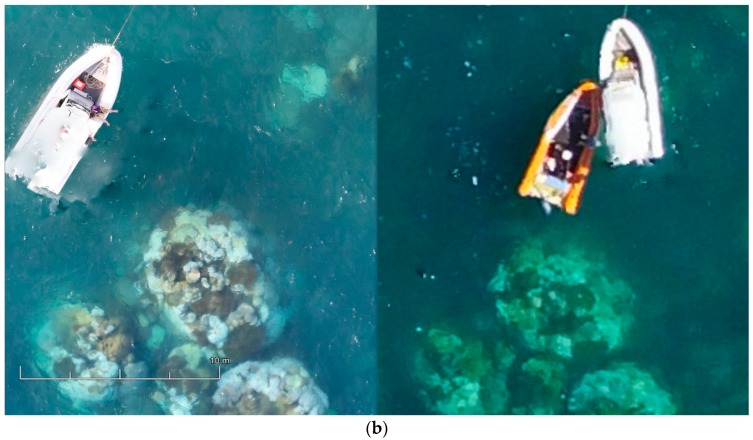
(**a**) Photogrammetry orthomosaic of western part of Pandora Reef (8 Ha) shows 400% zoomed fragments of bleaching events and the AIMS’s Antares research boat on the bottom right (tide height 1.6 m). (**b**) Fragments of the orthomosaics from Canon 5DS R (left) and DJI Phantom 3 Pro imagery (right).

Processing Canon 5DS R images gives significantly better 9.91 mm/px resolution of the orthomosaic/3D mode/DEMl, also bringing larger dynamic range to imagery (Figure 15b), and fully customised lenses and shooting options. 

Figure 15a shows an orthomosaic generated out of 395 50,6-megapixel Canon 5DS R images processed on medium quality settings, which resulted in a 1.33 gigapixel image (47,904 × 29,080) over an 8 hectare area of primary AIMS interest. These orthomosaics give researchers a method of monitoring benthic cover and geomorphological variances in benthic algae (Murfitt et al. [10]). Orthomosaics assist in identifying regions that are at greatest risk of degradation, and ascertain what the cause of this degradation is and what systems need to be employed to try and reduce/prevent further degradation in that region. The orthomosaics also give researchers a method for identifying in-water survey data points, and giving approximations of what coral is typically found in this region. For example, Palma et al. [11] found that small rocky outcrops with a regular shape were typically indicative of soft corals whereas large colonies in rocky regions typically indicated *Acropora* coral.

Airborne ultra-HD DSLR cameras provide researchers with a complex and fully adjustable tool resulting in very detailed snapshots of the marine environment. Larger dynamic range, higher resolution, more accurate focusing and white-balancing comes at the cost of a heavier and larger UAV to carry the sensor. We note also that other factors, such as correct camera settings and camera orientation relative to the sun and the object of interest, position of the sun, tide heights, sea state, and water torpidity, define the success, and sometimes are the most important factors for reef monitoring by remote sensing.

#### 4.1.2. Photogrammetry Ocean Floor Digital Elevation Models

**Figure 16:**
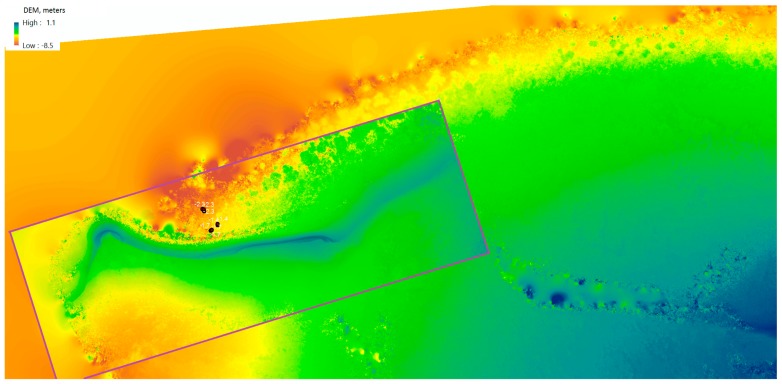
Digital elevation model (DEM) of Pandora Reef generated from Phantom 3 Pro and Canon 5DS R (purple area) and in-water survey depths (black dots).

Figure 16 shows the overlaid example of the DEM generated of the ocean floor surface of a fragment of Pandora Reef reconstructed out of Phantom 3 Pro (22 cm/px resolution) and Canon 5DS R (3.96 cm/px resolution) imagery. Despite the presence of minor artefacts and anomalies due to the presence of the moving border between two different mediums (sea and air), we observed adequate level of detail and accuracy of reconstruction for shallow waters (up to 5–8 m deep).

### 4.2. Spectral Signature Extraction Results

**Figure 17:**
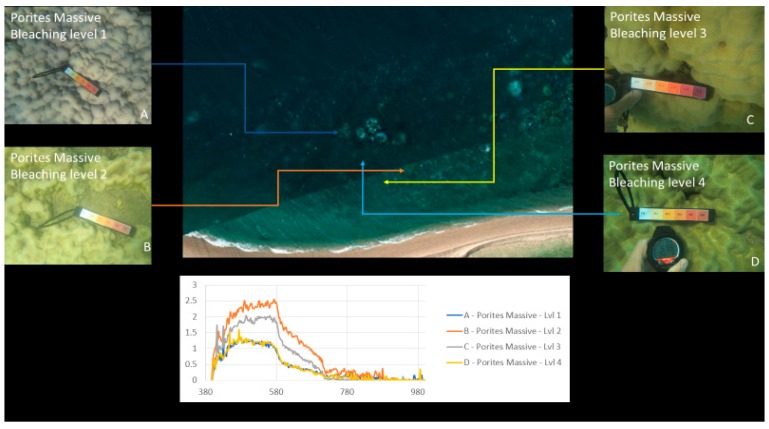
Example of spectral response “fingerprints” of bleaching levels 1 to 4 for *Porites* massive coral.

**Figure 18:**
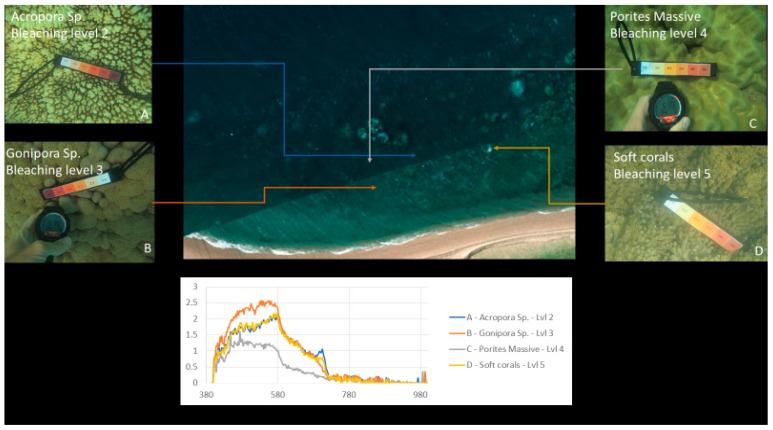
Example of spectral response “fingerprints” of bleaching levels 2, 3, 4 and 5 for *Acropora*, *Gonipora*, *Porites* massive and soft corals, respectively, for spectral comparison.

Figure 10, Figure 17 and Figure 18 show an example of spectral signature extraction for one coral type. Figure 18 shows the results with *Porites* massive bleaching level one and six, Figure 18 with soft corals level 5, *Porites* massive level 4, *Goniopora* level 3, and *Acropora* level 2. These spectral similarities will be one of the greatest potential causes of error in the classification step (step 9) and require processing of each identified point to confirm that the classification is correct. This will be done by solely comparing the classifications in the bands where differences are discernible.

### 4.3. Classification Results

**Figure 19:**
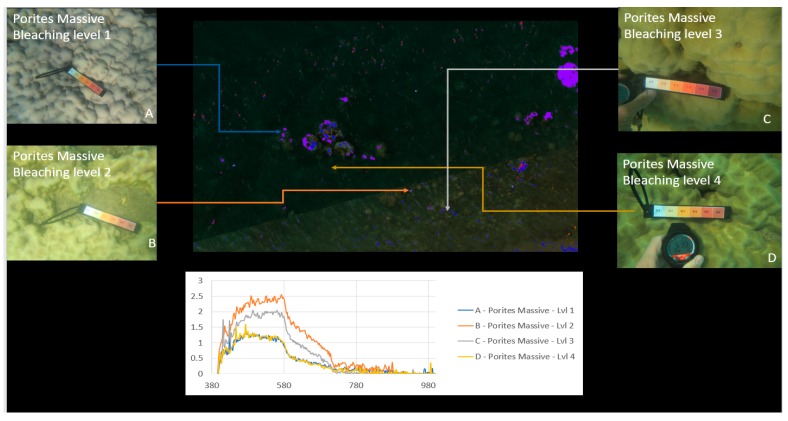
*Porites* massive material classification, with water classification omitted.

Material classification through Scyven, although very similar to ENVI, is done by taking the spectral fingerprint data (Figure 9, Figure 17 and Figure 18) and then running it through an automated process, which matches and groups sets of specific spectral data based on their similarity. Figure 19 shows the results of material classification applied to *Porites* massive coral with the other object classification omitted for clarity.

**Figure 20:**
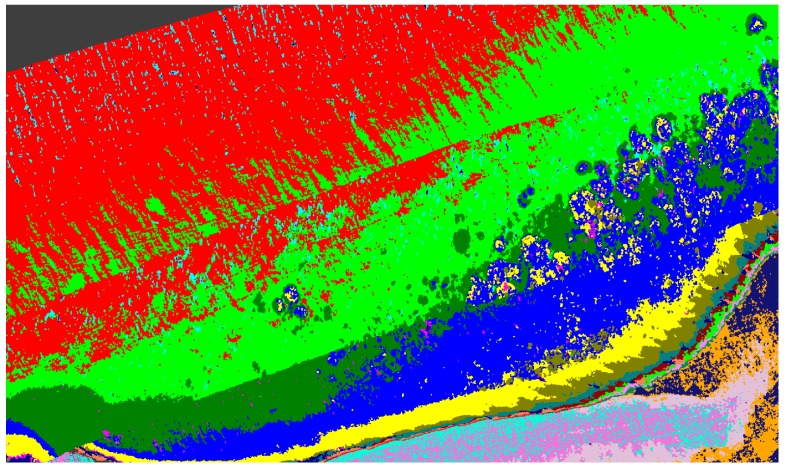
Unsupervised material classification for Pandora Reef.

Figure 20 shows output of the automatic unsupervised material classification from ENVI, to highlight and show variations which are typically associated with *Porites* massive coral indices (Table 1). These data are utilized to show regions that require exclusion for reduction of false positives.

**Figure 21:**
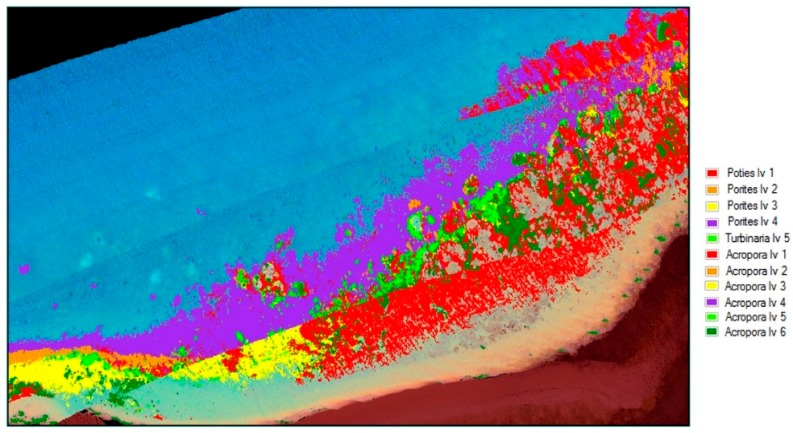
Support vector machine (SVM) material classification for Pandora Reef.

Once the data has been refined, it can then be classified using the extracted spectral signatures (step 7 Figure 1) and the reef indices generated (step 8 Figure 1), whilst also being careful and taking note of the regions that were highlighted by the unsupervised classifications. For example, in the case of Figure 20, the benthos is shown as light green, spume as light yellow, and the numerous additional misclassifications caused by the ill-classified sandbar. Figure 21 shows the results of this classification after refining the extracted spectral signatures using additional noise reduction algorithms in the form of spectral and spatial filtering, additional ground truth discrimination from unsupervised classification results, and removal of false positives highlighted by coral bleaching indices (Table 1) developed from Figure 20 and Table 2.

Table 3 Results of the accuracy assessment with labelled data material classification has been completed.

**Figure 22:**
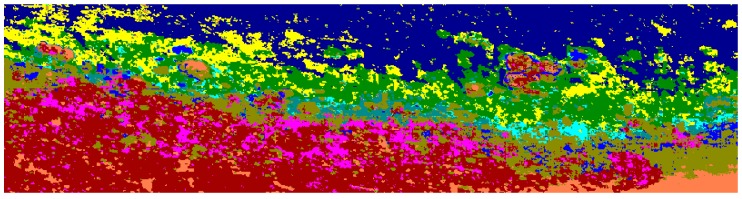
Support vector machine (SVM) material classification for Pandora Reef with spume regions marked in Figure 5 excluded.

The classification uses all the information in the image for classifying the different coral types. The accuracy results in Table 3 show the effect of including images with spume during the image processing phase. Classifications which have been greatly affected have been marked with EA (excessive area) in the table. Figure 22 shows the improved results of refining the data by excluding those regions of spume (marked in Figure 6), and this is reflected in Table 4.

### 4.4. Index Results

**Figure 23:**
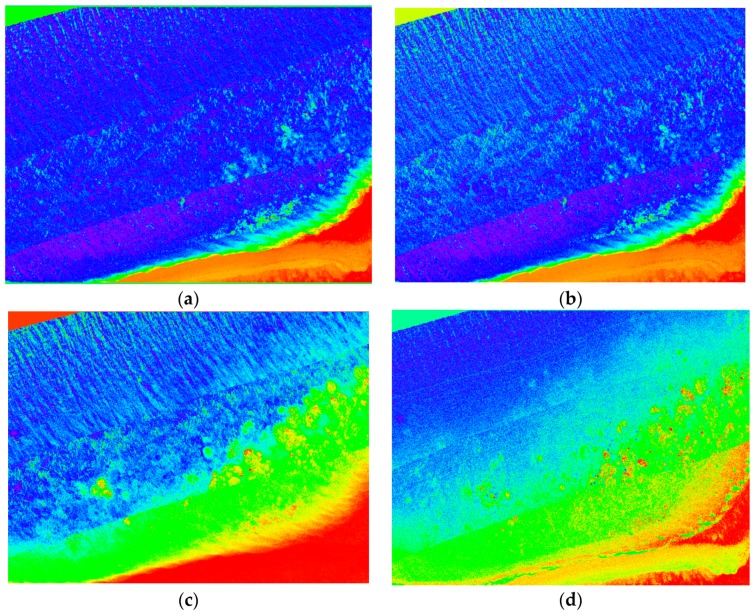
Full colour spectrum images blue to red intensity (red high), (**a**) NDVI yellow; (**b**) NDVI green; (**c**) *Turbinaria* index Tlv5.3; (**d**) *Acropora* index Alv1.2.

Different indices were computed in Section 2.8 in order to highlight the symptoms of bleaching across various genera of coral. Figure 23 shows examples of two NDVI images in the green and yellow wavelengths (identified by indices as bands with greatest variances over multiple bleaching levels), as well as two coral bleaching indices calculated from the hyperspectral data for the *Acropora* and *Turbinaria* genera of coral. This comparison shows the overall difference between the index output and the NDVI, where the NDVI images gives a clear representation, primarily focusing on the healthiest coral. The indices are capable of showing the overall health of the reef by not only showing the healthiest parts, but also all the various genera of coral at different levels of bleaching, and give health approximations based on benthic cover.

The results of the mean spectral signature extraction show a trend between spectral reflectance and severity of bleaching, which is consistent across various genera of coral. Coral bleaching indices developed from Figure 12 allow for analysis of stress caused by bleaching, however, these indices are not always able to distinguish coral genus from bleaching level, and may only be useful in monitoring temporal changes. NDVI images give a more easily interpreted representation, but cannot distinguish between variances of different genera and only show limited reef health data.

### 4.5. Limitations

The methodology shown in this manuscript has limitations, and requires careful and detailed planning of in-water and airborne surveys that can affect the overall feasibility and accuracy of the classification. The ability to classify objects can be greatly distorted or made near impossible, depending on water depth and the methodology used. Lesser and Mobley [12] found that it is possible to conduct coral classification with hyperspectral imagery to depths of around 10–12 m; this, however, requires the use of the Lambert–Beer law, which requires additional data of measured photoabsorption from testing areas. In this work, we use a band ratio equation which drops the maximum classification depth to around 6–8 m, which is satisfactory for large-scale remote sensing in either inaccessible or near-inaccessible areas, and offers options for health monitoring, however, this is a limitation for remote sensing of reef sections that do not fit these constraints.

Water turbidity, tidal conditions, and weather conditions all play a part in affecting the overall quality of the captured data. Water turbidity can affect the ability to identify coral in regions and, depending on its level, can prevent the ability to identify any coral. Nonetheless, data with moderate levels of turbidity still have some value in showing the current level of benthos/symbiotic algae in the region. Tidal conditions can cause significant amounts of spume to be created, methods can be employed to remove sections affected, but their effectiveness is limited by the spumes’ location and, where classification is required, can cause serious issues with the overall accuracy of results.

## 5. Conclusions

The results show that airborne UAV-based hyperspectral imagery has the potential to detect coral bleaching over large areas across multiple coral genera, where accurate visual inspection may not be possible. The methods and workflow developed during this study enabled material classification using both supervised and unsupervised methods to gain results with an acceptable percentage of error. The results presented and coral indices generated will contribute to the generation of valuable information for reef protection by offering methods that highlight possible symptoms of bleaching in a timely manner. It could also be extrapolated to other areas of research in remote sensing, such as mineral exploration, biodiversity, and ecological assessment.

## Figures and Tables

**Table 1:** Coral bleaching indices used in the analysis of reef type and bleaching.

Coral Index	Equation
Genus Classification	(R_540_ − R_575_/(R_450_ + R_586_)
Bleaching Classification	
*Acropora*	
Bleaching Level 1 (Alv1)	Alv1.1 = (R_395_ − R_404_)/(R_395_ + R_404_) Alv1.2 = (R_575_ − R_604_)/(R_575_ + R_604_) Alv1.3 = (R_711_ − R_732_)/(R_711_ + R_732_)
Bleaching Level 2 (Alv2)	Alv2.1 = (R_404_ − R_489_)/(R_404_ + R_489_) Alv2.2 = (R_595_ − R_662_)/(R_595_ + R_662_)
Bleaching Level 3 (Alv3)	Alv3.1 = (R_446_ − R_473_)/(R_446_ + R_473_) Alv3.2 = (R_531_ − R_555_)/(R_531_ + R_555_) Alv3.3 = (R_586_ − R_622_)/(R_586_ + R_622_)
Bleaching Level 4 (Alv4)	Alv4.1 = (R_446_ − R_489_)/(R_446_ + R_489_) Alv4.2 = (R_569_ − R_600_)/(R_569_ + R_600_) Alv4.3 = (R_611_ − R_671_)/(R_611_ + R_671_)
Bleaching Level 5 (Alv5)	Alv5.1 = (R_484_ − R_522_)/(R_484_ + R_522_) Alv5.2 = (R_695_ − R_720_)/(R_695_ + R_720_)
Bleaching Level 6 (Alv6)	Alv6.1 = (R_400_ − R_418_)/(R_400_ + R_418_) Alv6.2 = (R_460_ − R_484_)/(R_460_ + R_484_) Alv6.3 = (R_724_ − R_768_)/(R_724_ + R_768_)
*Porites* Massive	
Bleaching Level 1 (PLv1)	PLv1.1 = (R_437_ − R_473_)/(R_437_ + R_473_) PLv1.2 = (R_680_ − R_737_)/(R_680_ + R_737_)
Bleaching Level 2 (PLv2)	PLv2.1 = (R_411_ − R_473_)/(R_411_ + R_473_) PLv2.2 = (R_640_ − R_671_)/(R_640_ + R_671_)
Bleaching Level 3 (PLv3)	PLv3.1 = (R_429_ − R_473_)/(R_429_ + R_473_) PLv3.2 = (R_576_ − R_640_)/(R_576_ + R_640_)
Bleaching Level 4 (PLv4)	PLv4.1 = (R_406_ − R_418_)/(R_406_ + R_418_) PLv4.2 = (R_533_ − R_582_)/(R_533_ + R_582_)
*Gonipora*	
Bleaching Level 3 (GLv3)	GLv3.1 = (R_409_ − R_477_)/(R_409_ + R_477_) GLv3.2 = (R_640_ − R_722_)/(R_640_ + R_722_)
*Turbinaria*	
Bleaching Level 5 (TLv5)	TLv5.1 = (R_415_ − R_442_)/(R_415_ + R_442_) TLv5.2 = (R_471_ − R_486_)/(R_471_ + R_486_) TLv5.3 = (R_500_ − R_544_)/(R_500_ + R_544_) TLv5.4 = (R_675_ − R_717_)/(R_675_ + R_717_)
Soft Coral	
Bleaching Level 5 (SLv5)	SLv5.1 = (R_429_ − R_444_)/(R_429_ + R_444_) SLv5.2 = (R_506_ − R_544_)/(R_506_ + R_544_) SLv5.3 = (R_577_ − R_604_)/(R_577_ + R_604_) SLv5.4 = (R_662_ − R_708_)/(R_662_ + R_708_)

**Table 2:** Fragment of the attribute table at Pandora Reef (14 of 64 in-water data collected shown).

Photo ID	Coral Type	Lv Bleached	Bleached	Depth	Notes	Latitude	Longitude	Pixel *x*	Pixel *y*
76	*Porites massive*	1	Yes		1.5>	18.8129	146.4267	1489	1412
77	*Porites massive*	1	Yes			18.8129	146.4267	1507	1421
78	*Porites massive*	4	No	2.3		18.8130	146.4268	1544	1567
79	*Porites massive*	4	No	2.3		18.8130	146.4268	1529	1548
80	*Porites massive*	4	No	2.3		18.8130	146.4268	1541	1551
81	*Goniopora* sp.	3	No	1.7		18.8132	146.4268	1598	1733
82	*Goniopora* sp.	3	No	1.7		18.8132	146.4268	1604	1729
83	*Goniopora* sp.	3	No	1.7		18.8132	146.4268	1607	1727
84	*Acropora* sp.	2	Yes		Acropora plate.	18.8131	146.4268	1604	1669
85	*Acropora* sp.	2	Yes		Acropora plate.	18.8131	146.4268	1604	1666
86	*Acropora* sp.	3	No	1.4	Acropora plate.	18.8131	146.4269	1659	1684
87	*Acropora* sp.	3	No	1.4		18.8131	146.4269	1658	1675
88	*Porites massive*	3	No		1.5>	18.8131	146.4268	1610	1666
89	*Porites massive*	3	No			18.8131	146.4268	1615	1675
90	*Porites massive*	3	No		1.5>	18.8131	146.4269	1633	1636

**Table 3:** Material classification accuracy assessment for Figure 21 (EA = excessive area).

Coral Type	Bleaching Level	Signature Accuracy (%)	Points Found	Accuracy (%)	Found Pixels	Area (%)	Overall Accuracy (%)
*Porites massive*	1	79.17	2/3	66.667	91,157	2.932	66.67
*Porites massive*	2	94.44	1/2	50.00	30,007	0.965	50.00
*Porites massive*	3	68.75	2/7	28.571	55,529	1.786	28.57
*Porites massive*	4	100.00	4/5	80.00	339,305	10.915	0 (EA)
*Acropora* sp.	1	100.00	7/13	53.846	173,810	5.591	53.85
*Acropora* sp.	2	46.67	2/4	50.00	1248	0.040	46.67
*Acropora* sp.	3	100.00	2/2	100.00	38,729	1.246	100.00
*Acropora* sp.	4	100.00	2/2	100.00	41,618	1.339	100.00
*Acropora* sp.	5	100.00	3/14	21.428	32,119	1.033	21.43
*Acropora* sp.	6	100.00	2/2	100.00	115,879	3.728	0 (EA)
*Soft coral*	5	44.00	2/2	100.00	392,569	12.628	0 (EA)
*Turbinaria* sp.	5	73.91	2/2	100.00	2763	0.089	73.91

**Table 4:** Material classification accuracy assessment for Figure 22 at Pandora Reef where regions with spume region marker in Figure 5 are excluded.

Coral Type	Bleaching Level	Signature Accuracy (%)	Points Found	Accuracy (%)	Found Pixels	Area (%)	Overall Accuracy (%)
*Porites massive*	1	88.79	2/2	100.00	238,998	6.636	88.79
*Porites massive*	2	89.32	2/2	100.00	57,323	1.592	89.32
*Porites massive*	3	96.32	1/1	100.00	221,203	6.142	96.32
*Porites massive*	4	88.13	3/3	100.00	45,386	1.260	88.13
*Acropora* sp.	1	96.54	4/4	100.00	1,130,663	31.396	0 (EA)
*Acropora* sp.	4	93.54	2/2	100.00	55,085	1.530	93.54
*Acropora* sp.	5	90.75	3/3	100.00	477,663	13.264	0 (EA)
*Acropora* sp.	6	90.27	2/2	100.00	214,046	5.944	90.27

## References

[1] Hughes T., Kerry J., Baird A., Connolly S., Dietzel A., Eakin M., Heron S., Hoey A., Hoogenboom M., Liu G. (2018). Global warming transforms coral reef assemblages. Nature.

[2] (2016). AIMS.gov Resources: Coral Bleaching Events. http://www.aims.gov.au/docs/research/climate-change/coral-bleaching/bleaching-events.html.

[3] Anderson D., Armstrong R., Weil E. (2013). Hyperspectral Sensing of Disease Stress in the Caribbean Reef-Building Coral, *Orbicella faveolata*—Perspectives for the Field of Coral Disease Monitoring. PLoS ONE.

[4] Goodman J.A., Purkis S.J., Phinn S.R. (2013). Coral Reef Remote Sensing. A Guide for Mapping, Monitoring, and Management.

[5] Hamylton S., Hedley J., Beaman R. (2015). Derivation of High-Resolution Bathymetry from Multispectral Satellite Imagery: A Comparison of Empirical and Optimisation Methods through Geographical Error Analysis. Remote Sens..

[6] Gordon H.R., Brown O.B. (1974). Influence of bottom albedo on the diffuse reflectance of a flat homogeneous ocean. Appl. Opt..

[7] Mishra R D., Narumalani S., Rundquist D., Lawson M., Perk R. (2007). Enhancing the detection and classification of coral reef and associated benthic habitats: A hyperspectral remote sensing approach. J. Geophys. Res. Oceans.

[8] Zoffoli M., Frouin R., Kampel M. (2004). Water Column Correction for Coral Reef Studies by Remote Sensing. Sensors.

[9] Zanaty E. (2012). Support Vector Machines versus Multilayer Perception in data classification. Egypt. Inform. J..

[10] Grasmuek M., Eberli G., Viggano D., Correa T., Rathwel G., Lou J. (2006). Autonomous underwater vehicle (AUV) mapping reveals coral mound distribution, morphology, and oceanography in deep water of the Straits of Florida. Oceans.

[11] Murfitt S., Allan B., Bellgrove A., Rattray A., Young M., Lerodiaconou D. (2017). Applications of unmanned aerial vehicles in intertidal reef monitoring. Sci. Rep..

[12] Lesser M., Mobley C. (2007). Bathymetry, water optical properties, and benthic classification of coral reefs using hyperspectral remote sensing imagery. Coral Reefs.

